# Recycled Heavy Bio Oil as Performance Enhancer for Rubberized Bituminous Binders

**DOI:** 10.3390/polym11050800

**Published:** 2019-05-04

**Authors:** Jiangmiao Yu, Zhibin Ren, Zheming Gao, Qi Wu, Zihan Zhu, Huayang Yu

**Affiliations:** 1 School of Civil Engineering and Transportation, South China University of Technology, Wushan Road, Tianhe District, Guangzhou 510000, China; yujm@scut.edu.cn (J.Y.); mszhibinren@mail.scut.edu.cn (Z.R.); 201730187102@mail.scut.edu.cn (Z.Z.); 2 Sonny Astani Department of Civil and Environmental Engineering, University of Southern California, Los Angeles, CA 90089, USA; zhemingg@usc.edu; 3 Guangdong Province Communications Planning& Design Institute Co., Ltd, Xinghua Road, Tianhe District, Guangzhou 510000, China; wuqi614@126.com

**Keywords:** asphalt rubber, bio-asphalt, mixing sequence, workability, storage stability

## Abstract

Asphalt rubber (AR) is a sustainable paving material with merits including waste tire consumption, low traffic noise, and enhanced mechanical performance. However, the poor workability and storage stability limited its further application. This study attempted to alleviate these two concerns of AR simultaneously by incorporating heavy bio oil (HBO). To achieve this goal, bio-AR binders with three different mixing sequences were prepared. A series of rheological and chemical tests were conducted. Test results prove that the bio-AR binders exhibited superior rutting and fatigue resistance compared to AR binder. The viscosity values of bio-ARs were closed to AR modified with commercial warm mix additive, which indicates enhancement in workability. Due to the relatively high density of HBO, the density difference between the asphalt liquid phase and crumb rubber in the bio-AR system narrowed, which brought improved storage stability. Among bio-ARs prepared with different mixing sequences, the direct mixing one (ARB) had the most satisfied overall performance. The early incorporation of HBO had limited negative influence on binder performance, but allowed for more energy saving during the bio-AR binder production. Future study will be conducted on performance of bio-AR mixtures and quantitative estimation of its energy saving during the blending and compacting process.

## 1. Introduction

Asphalt mixture, composed of bituminous binder and mineral aggregates, is the most common paving material for highways and urban roads. The durability of asphalt pavement highly depends on the rheological properties of the bituminous binder. Due to the growing traffic loading and aggravation of axis, incorporating polymer modifiers (e.g., styrene-butadiene-styrene, crumb rubber, polyethylene) in virgin bitumen for superior adhesive and cohesive performance has become a common practice [1,2,3,4]. Among the modified bituminous binders, asphalt rubber (AR) is considered a green paving material with attractive engineering and environmental benefits. During its production, certain amount of crumb rubber particles (usually 15–20 wt %) are blended with hot virgin bitumen. Light fractions of bitumen are absorbed by the crumb rubber modifiers (CRM), which leads to the swelling of the crumb rubber [5,6]. AR contributes to the recycling of abundant disposal waste tires in an efficient and economical way [7]. The incorporation of resilient rubber particles also leads to enhanced rutting, fatigue, and low-temperature cracking resistance of asphalt pavement [8,9,10]. Moreover, the elastic rubber particles are beneficial for decreasing the traffic noise generated by the tire-road contact and abrasion [11].

Despite the previously mentioned metrics, two main concerns of AR, the poor workability, and storage stability limit its further application. The highly viscous behavior of the AR binder causes the workability concern. In recent years, warm mix asphalt (WMA), which is a clean production technology, has been used with AR to address the workability concern [12]. It is reported that a maximum decrement of 55 °C can be obtained by using chemical WMA additives, which certainly reduces the energy consumption and construction emission for the AR pavement [13]. The storage stability concern of AR is caused by the density difference between the incorporated CRM particles and the liquid bitumen. It is reported that nano-montmorillonite exhibited a significantly positive effect for reducing the segregation of CRM and the bitumen liquid phase of AR when storing in an elevated temperature [14]. However, very few practical approaches have been proposed for the improvement of both workability and storage stability of AR.

Recently, using bio-asphalt from bio-waste to replace part of the petroleum-based bitumen has been a hot topic for pavement researchers. It is reported that the heavy bio-oil can significantly decrease the viscosity of asphalt and bring better workability [15]. The common dosage of bio-oil is usually 20–40% by weight of bio-asphalt [16,17]. However, the bio-asphalt was found to have relatively poorer rutting and fatigue resistance. The bio-asphalt is not quite stable since a certain amount of bio-oil may evaporate during the construction process, which makes the asphalt content in the final asphalt pavement uncontrollable [18]. Considering the low viscosity and suitable density (1.07 g/cm^3^) of heavy bio oil (HBO), it is promising to use it as a performance enhancer for the AR binder, which may simultaneously alleviate the workability and storage stability concern of AR. In addition, when used as an additive rather than an alternate of bitumen, the dosage of HBO can be limited to 5–10% by weight of virgin bitumen, which makes the modified binder more stable compared to traditional bio-asphalt materials.

The objective of this study was to evaluate the feasibly of incorporating heavy bio-oil to improve both the workability and storage stability of the AR binder. To achieve this goal, rheological and chemical tests were conducted on modified bitumen. The virgin bitumen, CRM and HBO, were blended by three different mixing sequences. Physical and rheological properties, including penetration, softening point, Superpave rutting/fatigue factors, and low temperature stiffness of asphalt rubber modified with HBO (HBO-AR) binders were characterized. Gel permeation chromatography (GPC) analysis and Fourier Transform Infrared Spectroscopy (FTIR), which characterizes the molecular weight distribution and chemical functional groups respectively, were conducted to reveal the compound modification mechanism of CRM and HBO. This study is anticipated to provide useful information to pavement researchers who are interested in mitigating the workability and storage stability concerns of rubberized asphalt binders.

## 2. Materials and Methods 

### 2.1. Materials

Pen60/70, which was provided by Guangzhou Xinyue Transportation Technology Co., Ltd., Guangzhou, China, was selected as virgin bitumen to prepare AR and HBO-AR binders. The hot AR binder was prepared by high shear mixing (10,000 rpm) crumb rubber (40 mesh, provided by Huayi Rubber Co., Ltd., Dujiangyan, China) and virgin bitumen for 1 h at 176 °C, which allows the rubber particles to swell and dissolve in bitumen fractions. The CRM content was set as 18 wt % by weight of the hot AR binder, which is consistent with some previous publications [12,19,20].

The HBO additives were provided by a new energy company named Handan Zhenfei Trade Limited Company in the Hebei province, China. It is caramel viscous liquid (containing 3 wt % water) with a density of 1.07 g/cm^3^. The mass loss ratio of HBO after rolling thin film oven (RTFO) aging is 41.13%.

Three mixing sequences were applied to prepared rubberized bituminous binder containing HBO. For all warm asphalt rubber (WAR) binders, the HBO or Evotherm-DAT (provided by MeadWestvac Company, Richmond, VA, USA) content was set as 5 wt % by weight of the hot AR binder. Specifically, AR-B refers to the WAR prepared by a conventional sequence, mixing HBO and the hot AR binder for 10 min at 160 °C. ARB was prepared by directly blending the same amount of virgin bitumen, CRM and HBO together at 160 °C for 60 min. BR-A used a pretreatment process in which CRM were soaked into liquid HBO for 48 h. This makes the HBO additive completely absorbed by the CRM particles. After that, the CRM containing HBO (B-CRM) was incorporated to virgin bitumen by 1 hour high shear mixing at 160 °C. For all rubberized binders with HBO, the mass ratio was set as 61:220:1000 for HBO, CRM, and virgin bitumen, respectively. In addition, the WAR binder with Evotherm-DAT (labelled as ER-A) was prepared as a control group to evaluate the performance of the HBO additive. ER-A is prepared by the pretreatment method like BR-A. According to Yu’s study, the pretreatment method provides the optimal performance for the WAR binder with Evotherm-DAT [20].

### 2.2. Testing Program

#### 2.2.1. Standardized Performance Tests

The penetration and softening point test were conducted to evaluate the general performance of test binders. Rotational viscosity was conducted for workability characterization at three different temperatures (135, 160, and 176 °C), using a Brookfield rotational viscometer (AMETEK Brookfield Company, Middleboro, MA, USA) with 20# spindle. A dynamic shear rheometer (DSR, Malvern Kinexus Lab+, Malvern analytical Company, UK) was used to conduct rheological tests.

The high temperature performance of prepared binders was characterized by both the Superpave rutting factor (G^*^/sin δ, for both unaged and RTFO aged samples) and non-recoverable creep compliance (J_nr_, for RTFO aged samples only). The standard RTFO procedure takes unaged asphalt binder samples in cylindrical glass bottles and places these bottles in a rotating carriage within an oven. The carriage rotates within the oven while the 163 °C temperature ages the samples for an additional 85 min. The G^*^/sin δ test started at 64 °C, and the temperature was increased automatically to the next PG temperature if the measured rutting factor was larger than the values, i.e., 1.0 kPa for unaged binder and 2.2 kPa for the RTFO binder. J_nr_ was evaluated by the multiple stress creep recover (MSCR) test at 64 °C. During the test, a creep load was applied for 1 s followed by 9 s of a recovery. Each specimen was subjected to 10 cycles with a creep stress of 0.1 kPa, which is followed by 10 cycles with a creep stress of 3.2 kPa.

The fatigue resistance was evaluated by the Superpave fatigue factor (G^*^sin δ) test and linear amplitude sweep (LAS) test with pressurized aging vessel (PAV) aged specimens. During the PAV aging procedure, the RTFO aged asphalt was placed in a chamber with 100 °C and 2.1 MPa for 20 h. The G^*^sin δ test was started at 25 °C with a decrement of 3 °C until the G^*^sin δ value was larger than 5000 kPa. The LAS test was started with a frequency sweep followed by a linear amplitude strain sweep to determine the cycles to failure denoted as *N_f_*. The fatigue failure is defined as the 35% reduction of the initial modulus in the LAS test, according to the viscoelastic continuum damage (VECD) model.

The bending beam rheometer (BBR, CANNON Instrument Company, State College, PA, USA) test was conducted for a low temperature performance evaluation. It characterized the stiffness and m-value of the test binder at a PAV aging state. The tests were implemented in a fluid bath with a constant load (980 ± 50 mN) at −12 °C, −18 °C, and −24 °C, respectively.

#### 2.2.2. Frequency Sweep

In addition to the standardized performance tests, the overall rheological performance of test binders was evaluated by master curves of a complex shear modulus (G^*^) based on the time-temperature superposition principle. Master curves (reference temperature: 60 °C) were obtained through a series of frequency sweeps at a temperature range from 76 to 4 °C with various frequencies between 0.01 and 30 Hz. The best fit of frequency sweep test data was conducted to obtain a single master curve based on the Williams-Landel-Ferry (WLF) formula (Equation (1) and Equation (2)) and the sigmoidal function (Equation (3)) [21].

(1) $$\log(a(T))=\frac{-C_{1}\Delta T}{C_{2}+\Delta T}$$

where a(T) is the shifting factor at one specific temperature *T*, Δ*T* is the different value between the test temperature and reference temperature, and *C*_1_ and *C*_2_ are model constants.

(2) log(ξ)=log(f)+log(a(T))

where *ξ* is the reduced frequency at reference temperatures and *f* is the test frequency at specific temperatures.

(3) $$\log(G^{*})=\delta +\frac{\alpha }{1+e^{\beta +\gamma \log(\xi )}}$$

where *β*, *γ* are the shape parameters, *α* is the span of G^*^ values, and δ is the minimum modulus value.

#### 2.2.3. Storage Stability Test

The storage stability was conducted to evaluate the phase separation of modified binders during the storage process at an elevated temperature. A lab-simulated high-temperature storage process was conducted on test binders. About 70 g of hot asphalt binder was first poured into an aluminum tube, which were then vertically stored at 163 °C for 48 h. The tube was then cooled down and cut into three equal parts horizontally. The top and bottom parts were used to identify their difference in property after storage. According to ASTM D36, the storage stability was characterized by the softening point difference between the binders in top and down parts.

#### 2.2.4. Chemical Tests

For a mechanism investigation, Gel Permeation Chromatography (GPC, Agilent 1260, Agilent Technologies Inc., Santa Clara, CA, USA) and Fourier transform infrared spectroscopy (FTIR, VERTEX 70, Bruker, Hamburg, Germany) were performed on prepared binders. The GPC test was conducted to evaluate the effect of crumb rubber and HBO on molecular weight distribution of liquid asphalt fractions, while FTIR tests characterized the chemical bonds and functional groups of test binders. 

For GPC tests, the test binders were dissolved in Tetrahydrofuran (THF) solvent and then filtered through a 0.45 μm Polytetrafluoroethylene (PTFE) syringe filter. The insoluble particles were removed by this process, while all the residual fractions can be completely dissolved in THF. By the filtering process, about 18–20 wt % of samples was filtered out. The asphalt-THF solution was drained through columns and allowed to flow at a rate of 0.5 ml/min, and the temperatures of the columns were maintained at 40 °C.

For FTIR tests, the test binder was first pressed to pellets with a thickness of approximately 1 mm, and then placed in a transmission holder and scanned. Infrared spectroscopy ranging from 4000 to 400 cm^−1^ was obtained by scanning using an FTIR spectrometer. 

The detailed information of conducted tests in this study was summarized in Table 1. For all the above-mentioned tests, three replicates were prepared and tested.

## 3. Results and Discussion

### 3.1. Penetration and Softening Point

**Figure 1:**
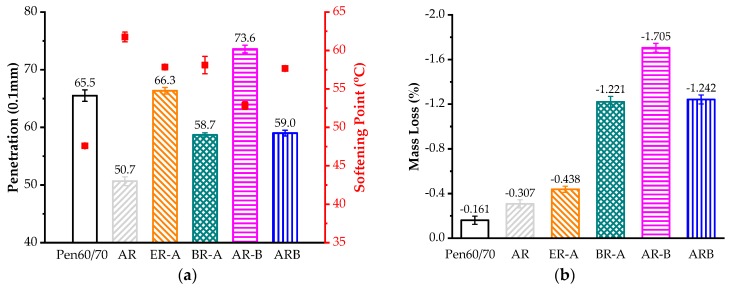
Conventional tests: (**a**) Penetration (histogram) and softening point (scatter diagram). (**b**) Mass loss after short-term aging.

Figure 1a shows the results of penetration and softening point tests. It is observed that CRM led to a lower penetration value and a higher softening point, which indicates that the rubberized binders were stiffer and more stable in an elevated temperature compared to virgin bitumen. The liquid additives, both HBO and Evotherm-DAT, made the rubberized binders softer and more sensitive to temperature variation. The effect of mixing sequence on penetration of the HBO-AR binder was clear. The one with conventional mixing order, AR-B, had even a higher penetration value than Pen60/70. By comparison, BR-A and ARB exhibited similar performance to ER-A, which is the WAR binder with a commercial WMA additive. One possible explanation is that the blending time of the AR binder and HBO additive (10 min) is not enough for a completed reaction among the components. It is also observed that the mass loss of AR-B after RTFO aging was higher than other WAR binders (Figure 1b).

### 3.2. Workability

**Figure 2:**
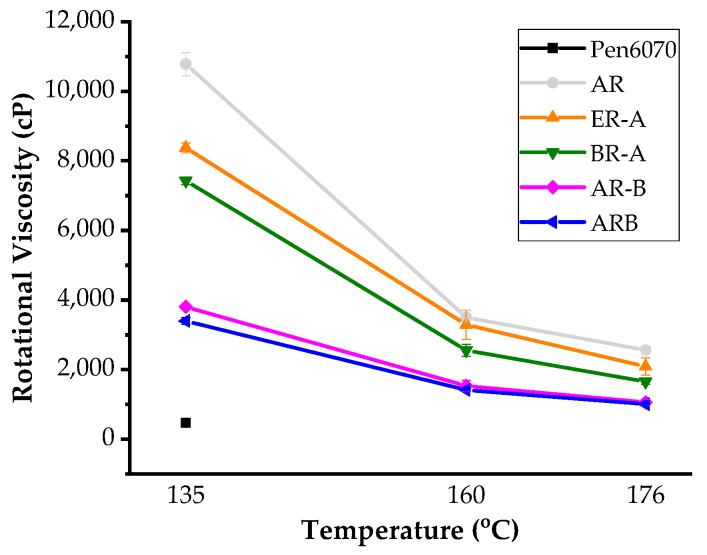
Rotational viscosity test results.

Figure 2 shows the rotational viscosity values of test binders at 135, 160, and 176 °C. The poor workability is the most critical concern for rubberized binders. As depicted, the viscosity of AR at 135 °C is almost 25 times that of Pen60/70. With the aid of liquid WMA additives, the WAR binders showed better workability, but the viscosity values were still higher than virgin bitumen. It is observed that HBO-AR binders had superior workability compared to ER-A binder, regardless of the mixing sequence. Consistent with the previous studies [20], the pretreatment sequence led to the poorest workability of HBO-ARs, while specimens prepared with direct mixing and conventional mixing methods had similar viscosity values. According to the AASHTO standard, mixtures with ARB and AR-B can be compacted at 160 °C since their viscosity values were lower than 3000 cP [22].

### 3.3. Rutting Resistance

**Figure 3:**
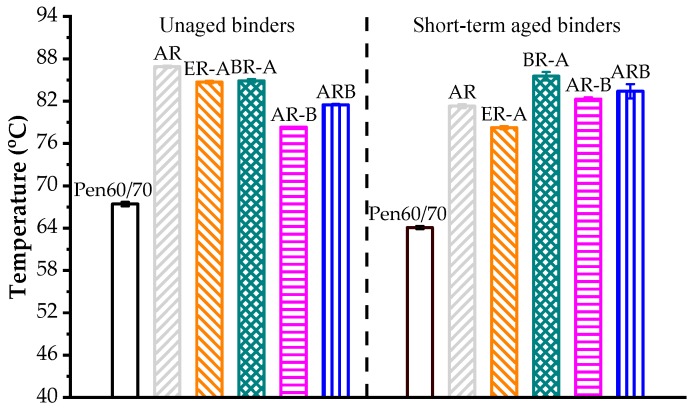
Superpave rutting factor results.

Figure 3 shows the results of the Superpave rutting factor test. As expected, CRM had a significant effect on enhancing the rutting resistance. In the unaged state, AR had the highest failure temperature. However, after short-term aging, the HBO-ARs showed better rutting performance than AR. Moreover, it is noted that all HBO-ARs outperformed ER-A in terms of rutting resistance. Among the HBO-AR binders prepared with different blending sequences, BR-A had the best high-temperature performance followed by ARB and AR-B.

Table 2 presents the MSCR test results. The J_nr_ difference of all modified binders did not meet the requirement of AASHTO TP70-13 i.e., < 75%. This is attributed to the extremely low J_nr_ values at the applied stress of 0.1 kPa [19]. Based on the J_nr_3.2 values, Evotherm-DAT had a negative effect on the rutting resistance while HBO showed a positive influence. Consistent with the results of the Superpave rutting factor test, BR-A exhibited the lowest J_nr_3.2 value, which indicates the best rutting resistance among three HBO-AR binders.

### 3.4. Fatigue Resistance

**Figure 4:**
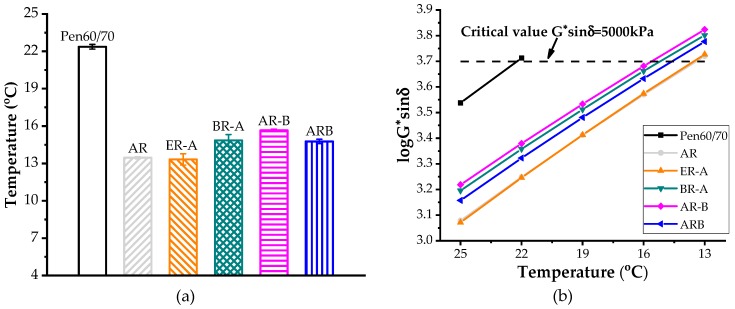
Fatigue performance of asphalt binders: (**a**) failure temperatures and (**b**) logarithm of G^*^sin δ values.

Figure 4a shows the fatigue failure temperatures and Figure 4b shows the relationship between the G^*^sin δ value and the test temperature. AASHTO M320 specified that the fatigue factor, G^*^sin δ, should be less than 5 MPa to pass a performance grade test at a specific temperature. The higher the fatigue failure temperature is, the poorer the fatigue resistance the test binder has. As depicted in Figure 4a, the failure temperature of AR was 8.9 °C lower than that of Pen60/70, which indicates superior fatigue resistance brought by CRM. The incorporation of Evotherm-DAT further enhanced the fatigue resistance by decreasing the failure temperature by 0.2 °C. By comparison, HBO had a negative effect on the fatigue performance of the rubberized binder. The failure temperature values of HBO-ARs were 1.3–2.2 °C higher than that of AR.

**Figure 5:**
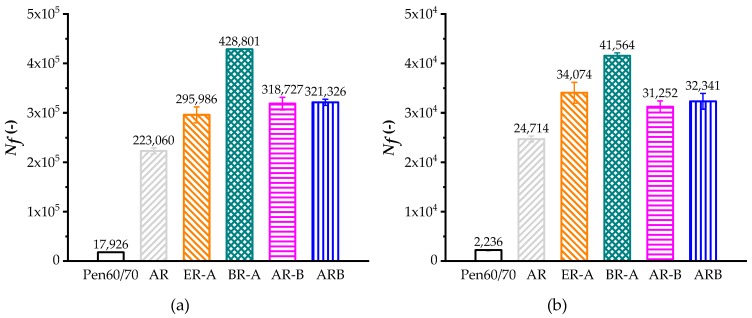
LAS test results: (**a**) applied strain of 2.5% and (**b**) applied strain of 5.0%.

**Figure 6:**
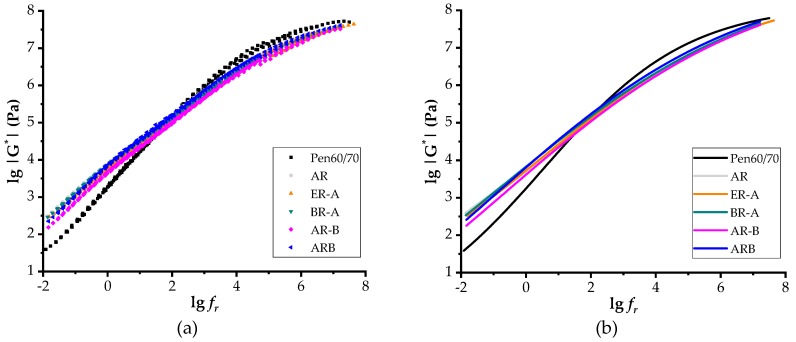
Master curves of test binders: (**a**) scatters of test results and (**b**) sigmoidal fitting curves.

Figure 5 shows the LAS test results at two applied strains (2.5% and 5%). Higher cycles to fatigue (*N_f_*) refer to better resistance of fatigue cracking. According to Figure 6, the *N_f_* values of AR were more than 11 times of virgin bitumen at 2.5% and 5% strain levels. Inconsistent with Superpave fatigue test results, the HBO-ARs had higher fatigue lives compared to AR based on LAS evaluation. Since previous studies have proven that LAS is a more reliable fatigue characterizing methods for bituminous specimens, it is believed that the HBO additive has a limited negative effect on the fatigue performance of AR [9,23].

### 3.5. Low Temperature Cracking Resistance

Table 3 shows the stiffness and m-values of test binders obtained by the BBR test. According to AASHTO T313, at one specific temperature grade, the m-value should be over 0.3, and the stiffness value should be less than 300 MPa. Higher stiffness values result in low-temperature cracking. According to Table 3, it is noted that all rubberized binders had lower stiffness compared to Pen60/70 at a low temperature, which is attributed to the resilient behavior of the CRM particles. According to thermal characterization, the glass transition temperatures of crumb rubber, HBO, and virgin asphalt are approximately −50 °C, −40 °C, and −25 °C (PAV aged binder), respectively. The incorporation of Evotherm-DAT and HBO exhibited a negative effect on the low-temperature performance of AR. Among the HBO-AR binders, the low temperature performance of ARB was slightly better than BR-A and AR-B.

### 3.6. Overall Rheological Behavior

Master curves were drawn to evaluate the overall rheological properties of asphalt binders at a wide angle of frequencies (10^−2^–10^−8^ Hz). To obtain the master curves, a series of complex mathematical calculations were conducted. The WLF formula (Equation (1) and Equation (2)) was first substituted into the sigmoidal function (Equation (3)) as Equation (4).

(4) $$\log(G^{*})=\delta +\frac{\alpha }{1+e^{\beta +\gamma (\log(f)+\frac{-C_{1}\Delta T}{C_{2}+\Delta T})}}$$

Then a nonlinear surface fit was conducted to obtain parameters *C*_1_ and *C*_2_ with log(*f*) and Δ*T* as independent variable using Equation (4). Different loading frequencies were shifted at a given temperature (60 °C) to obtain a single master curve based on Equation (2). Lastly, the fitting master curves were obtained by a best fit based on Equation (3). The parameters of the WLF formula and sigmoidal function were presented in Table 4.

Figure 6 shows the master curves of G^*^ of test binders at the reference temperature of 60 °C. Based on sigmoidal function, scatters and smooth curves were obtained in term of the lg|G^*^| versus reduced frequency (lg*f_r_*). Based on the time-temperature superposition principle of viscoelastic material, low frequency refers to high temperature and vice versa. As expected, the increased frequency resulted in an increase of complex shear modulus. According to Figure 6b, the moduli of all rubberized binders were lower in high frequencies but higher in low frequencies compared to neat bitumen, which indicates superior performance in both high and low temperatures. Findings obtained from master curves were consistent with the results obtained by the Superpave rutting factor test, the MSCR test, and the BBR test.

### 3.7. Storage Stability

**Figure 7:**
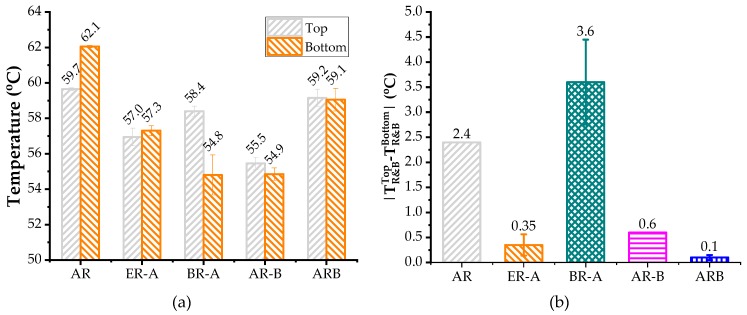
Softening point difference of test binders after storage: (**a**) Softening points of top and bottom sections. (**b**) D-value between top and bottom sections.

Figure 7 show the softening point difference (D-value) of test binders after lab-simulated storage. A smaller D-value refers to better storage stability. According to Figure 7, both AR-B and ARB exhibited better storage stability compared to AR, while BR-A had poorer performance. Since HBO had higher density compared to virgin bitumen, it narrowed the density difference between the bitumen liquid phase and CRM particles in the bio-AR system, which provided resistance to prevent the settling of CRM particles. However, the reason why the poorest storage stability belonged to BR-A still required further investigation.

### 3.8. Molecular Weight Distribution

**Figure 8:**
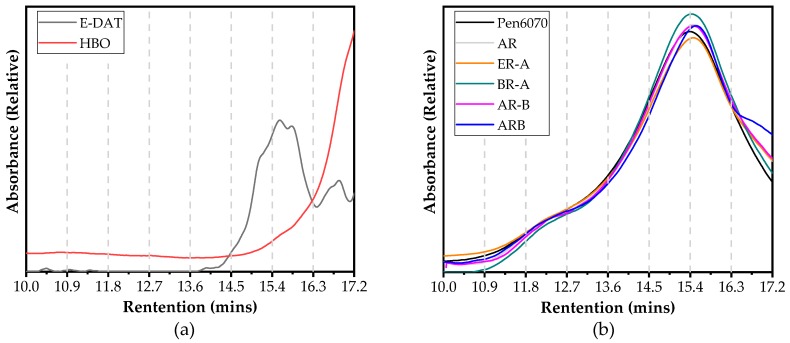
GPC test results: (**a**) chromatograms of Evotherm-DAT and HBO and (**b**) chromatograms of test binders.

Figure 8 presents the GPC test results of liquid additives and test binders. The elution amount of specific molecular range can be obtained by analyzing the curves’ fluctuation of test binders. According to their molecular weight, the constituents of all specimens were classified into several groups [24,25,26,27]. As shown in Figure 8, the chromatograms were divided into eight slices based on the selected retention time ranging from 10 to 17.2 min (referring to molecular weight ranges from 48,386 to 245 g/mol). The entire area of eight slices for each specimen was adjusted to 1, and then the area ratio was used to compare their molecular weight distribution. A higher area ratio represents a larger percentage of the specific molecular size. It is shown in Figure 8a that the constituents of Evotherm-DAT gathered from 14.5 to 17.2 min, while those of HBO concentrated in 16.3 to 17.2 min. This indicates that the average molecular weight of HBO was much smaller than that of Evotherm-DAT. As shown in Figure 8b, the highest area ratio of ARB in 16.3 to 17.2 min may lead to the lowest average molecular weight among all binders.

Table 5 presents the GPC test results based on numerical statistics analysis. Four different parameters were selected to describe the variations of molecular weight distribution during the modification process, i.e. peak molecular weight (M_p_), number-average molecular weight (M_n_), weight-average molecular weight (M_w_), and polydispersity (PDI = M_w_/M_n_). It is noted that the PDI values of HBO and Evotherm-DAT were lower than that of asphalt binder samples, which indicates more concentrated distribution of molecular weight. AR had a lower M_n_ value but higher M_w_ value than Pen 60/70, which is likely caused by the modification of CRM dissolved in asphalt fractions. This indicates that the dissolution of CRM varied the molecular weight distribution slightly. Among three preparation procedures, the PDI of AR was 1.8068 and 1.3126 higher than those of BR-A and AR-B, respectively, while 0.2173 was lower than that of ARB. However, the M_w_ values of all HBO-ARs were lower than those of AR. Therefore, different preparation procedures led to a different molecular weight distribution. Yet, the large molecules’ content of all HBO-ARs was lower than that of AR. By contrast, incorporating Evotherm-DAT decreased both M_w_ and M_n_ of AR. One possible reason is that the dissolution process of CRM was promoted by the incorporation of Evotherm-DAT, which affected the molecular weight distribution significantly.

### 3.9. Chemical Bonds Variation

**Figure 9:**
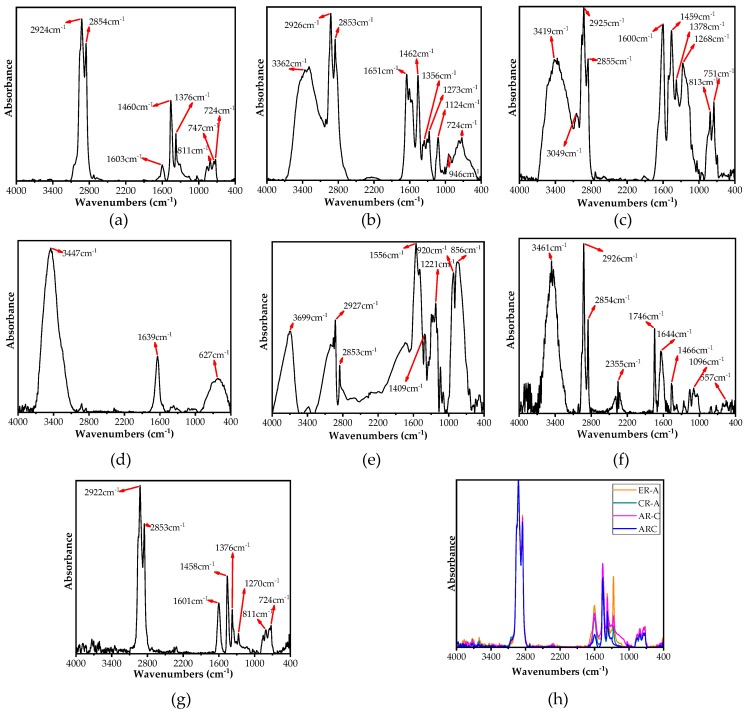
FTIR test results: (**a**) Base binder, (**b**) Evotherm–DAT, (**c**) HBO, (**d**) CRM, (**e**) E–CRM, (**f**) B–CRM, (**g**) AR, and (**h**) Evotherm–DAT and HBO modified binders.

Figure 9a–c present the FTIR spectra of virgin bitumen, Evotherm-DAT, and HBO, respectively. These three kinds of materials have similar elementary composition (including carbon, hydrogen, oxygen, nitrogen, and sulfur), but display quite a difference in their functional groups. Evotherm-DAT and HBO show more complex chemical composition compared to neat bitumen [28]. Evotherm-DAT peaked at approximately 3362 cm^−1^ (O-H or N-H stretching vibration of the hydrogen-bonded hydroxyl group and amino groups), 1651 cm^−1^ (C=O stretching vibration of secondary amides), and 1356 cm^−1^ (symmetric SO2 stretching of sulphone group), which indicated the existence of amines, amino ions, and sulfur-containing organics, respectively [29]. As shown in Figure 9c, it is noted that HBO peaked at 3417 cm^−1^ (O-H or N-H stretching vibration of hydrogen-bonded hydroxyl group and amino groups), 3049 cm^−1^ (=CH stretching vibration of the benzene ring), and 1600 cm^−1^ (C=C stretching vibration of the aromatic ring). These absorbance peaks were most likely caused by the presence of phenol, cresol, and xylenol. HBO did not peak at 724 cm^−1^ (–(CH2)_n_– rocking vibration of alkane groups, n > 4). This may lead to a smaller average molecular weight than neat bitumen and Evotherm–DAT, which is consistent with the results of the GPC test.

**Figure 10:**
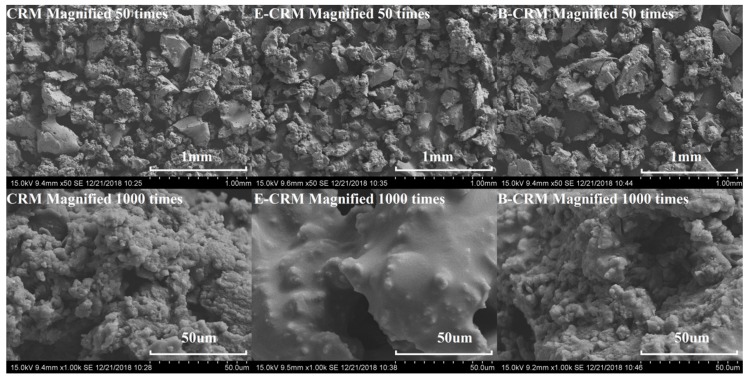
SEM results: variations of CRM before and after soaking in Evotherm–DAT or HBO.

Figure 9d–f show the FTIR spectra of CRM, CRM containing Evotherm–DAT (E–CRM) and B–CRM, while Figure 10 show their morphologies in micro scale (scanning electron microscope (SEM) images). E–CRM had a smoother surface than CRM, while B–CRM had no significant variation. As shown in Figure 9e, the spectra of E–CRM included most peaks found in CRM and Evotherm–DAT. However, the peaks occurring at 800 to 1600cm^−1^ showed up in the spectra of E–CEM compared to that of both CRM and Evotherm–DAT. This indicates the decrease of the –COOH group of Evotherm–DAT and the –OH group of rubber caused by a chemical reaction between the two groups. Approximately, it can be seen in Figure 9f that CRM and HBO can find similar peaks with B–CRM. Meanwhile, peaks at 1096 and 1746 cm^−1^ (C–O–C and C=O stretching) were observed in B–CRM, which did not occur in either CRM or HBO. This may demonstrate the chemical reaction between the phenol of HBO and residual acetone of CRM during its regenerative process.

The FTIR spectra of AR were shown in Figure 9g. The major absorption bands of AR occurred at similar locations with those of neat bitumen. However, adding CRM enhanced the absorbance by 700–1700cm^−1^ (mainly C–H stretching) and weakened the absorbance of 1639 cm^−1^ (C=O stretching). One possible reason is that the unsaturated functional groups of CRM and base binder was oxidized by high shear mixing. Therefore, the modification of CRM is not a single physical process. The modification can also result in the variation of functional groups and chemical bonds. As shown in Figure 9h, it is noted that all HBO–ARs and ER–A had very similar spectra of FTIR, which indicates their close chemical components [30]. 

The mechanism of HBO contributing to the modification of AR can be illustrated as follows. During the modification process through high shear mixing, chemical bonding and physical absorbing may result in redistribution of hydrocarbon chains [31,32]. Then one more compacted and steadier micro structure was obtained. The heavy bio oil, which is mainly composed by aromatics (phenol, cresol, and xylenol), will surround rubber particles. This will promote the devulcanization and depolymerization of crumb rubber [30], and eventually results in superior rheological properties of the modified binder.

## 4. Conclusions

This study evaluated the feasibly of using heavy bio–oil to improve both the workability and storage stability of the AR binder. Rheological and chemical tests were conducted to characterize the effects of HBO and its different preparation procedures for modifying AR. According to test results, the findings were obtained as follows.

- Compared to a conventional AR binder, the bio–ARs had superior rutting and fatigue resistance but slightly poorer low temperature performance.
- The bio–ARs exhibited better performance in both workability and storage stability compared to AR. Specifically, the warm mix effect of HBO additive is comparable to the commercial liquid WMA additive.
- The methods that incorporate bio–oil in earlier stages (direct mixing and pretreatment methods) had a very marginal negative effect on the performance of bio–AR. Moreover, they are more sustainable since they reduce both the temperature of the mixing AR binder with aggregate, and that of blending CRM with virgin asphalt.

The findings of this study have proven that HBO is a promising modifier that simultaneously alleviates enhancing the workability and storage stability of AR. Future study will focus on effect of HBO on AR mixtures and quantitative estimation of energy saving during blending and the compacting process.

## Figures and Tables

**Table 1:** Details of laboratory tests.

Performance	Experiments	Aging Level	Specification/Standard	Notes
**Conventional property**	Softening point	Unaged	ASTM D36	N/A
	Penetration		ASTM D5	25 °C
**Workability**	Rotational viscosity	Unaged	AASHTO T316	135, 160, and 176 °C
**Rutting resistance**	Rutting factor (G^*^/sin δ)	Unaged & RTFO- aged	AASHTO M320	beginning at 64 °C, 25 mm plate, 2 mm gap
	MSCR	RTFO- aged	AASHTO MP19-10	64 °C, 25-mm plate, 2-mm gap
**Fatigue resistance**	Fatigue factor (G^*^sin δ)	RTFO- + PAV- aged	AASHTO M320	beginning at 25 °C, 8-mm plate, 2-mm gap
	LAS		AASHTO TP101	25 °C, 8-mm plate, 2-mm gap
**Low temperature cracking resistance**	BBR	RTFO- + PAV- aged	AASHTO T313	−12, −18, −24 °C
**Overall rheological properties**	Frequency sweep	Unaged	N/A	4 to 76 °C, 0.01 to 30 Hz
**Storage stability**	Softening point	unaged	ASTM D36	N/A
**Mechanism investigation**	FTIR	unaged	N/A	N/A
	SEM		N/A	N/A
	GPC		GPC testing manual	30 °C

**Table 2:** MSCR test results.

Sample ID	J_nr_			% Recovery	
	0.1 kPa (kPa^−1^)	3.2 kPa (kPa^−1^)	J_nr_% Diff	0.1 kPa (kPa^−1^)	3.2 kPa (kPa^−1^)
**Pen60/70**	4.514 ± 0.166	5.007 ± 0.154	11.0 ± 0.6	0.7 ± 0.4	−0.5 ± 0.2
**AR**	0.186 ± 0.015	0.529 ± 0.002	186.4 ± 24.4	72.1 ± 2.0	35.0 ± 0.4
**ER-A**	0.087 ± 0.026	0.870 ± 0.044	954.5 ± 367.1	89.6 ± 3.3	26.1 ± 0.9
**BR-A**	0.041 ± 0.002	0.167 ± 0.013	307.2 ± 11.0	86.4 ± 0.8	54.0 ± 1.3
**AR-B**	0.123 ± 0.036	0.332 ± 0.092	171.7 ± 6.6	76.6 ± 2.9	46.4 ± 5.9
**ARB**	0.186 ± 0.039	0.399 ± 0.039	117.2 ± 25.1	65.7 ± 4.7	35.4 ± 2.2

The numbers after “±” are standard deviations.

**Table 3:** BBR test results.

Sample ID	−12 °C		−18 °C		−24 °C	
	Stiffness (MPa)	m-Value (× 10^−2^)	Stiffness (MPa)	m-Value (× 10^−2^)	Stiffness (MPa)	m-Value (× 10^−2^)
**Pen60/70**	277 ± 4.2	29.1 ± 1.5	534 ± 9.9	20.4 ± 0.6	N/A	N/A
**AR**	93.6 ± 14.7	37.9 ± 0.8	168 ± 12.7	32.2 ± 1.5	375 ± 24.0	20.4 ± 1.7
**ER-A**	183 ± 12.7	63.7 ± 18.2	311 ± 4.9	32.8 ± 7.9	484 ± 9.2	19.4 ± 1.8
**BR-A**	194 ± 17.7	49.2 ± 2.3	271 ± 7.8	24.7 ± 4.3	453 ± 62.9	19.8 ± 1.1
**AR-B**	112 ± 14.8	32.8 ± 1.1	243 ± 42.4	27.4 ± 2.0	448 ± 24.7	19.1 ± 1.6
**ARB**	97.0 ± 12.8	34.6 ± 3.8	208 ± 17.0	28.0 ± 3.6	412 ± 13.4	19.4 ± 0.9

The numbers after “±” are standard deviations.

**Table 4:** Model parameters of the WLF formula and sigmoidal function.

Parameters	WLF Formula		Sigmoidal Function				
	C_1_ (-)	C_2_ (-)	δ (Pa)	α (Pa)	B (-)	γ (-)	R^2^ @|G^*^| (-)
**Pen60/70**	−8.82557	138.09993	−0.55919	8.71694	0.25486	−0.45159	0.99942
**AR**	−8.26021	136.30605	−0.11497	8.67516	0.18174	-0.32416	0.99930
**ER-A**	−6.80041	117.83596	−0.44963	9.09011	0.16465	−0.30914	0.99887
**CR−A**	−8.10492	135.16876	−2.27332	11.3535	−0.14977	−0.24744	0.99926
**AR−C**	−7.69053	131.04835	−2.8193	11.86402	−0.17005	−0.25139	0.99925
**ARC**	−8.08466	132.99088	−2.87974	11.86654	−0.25577	−0.25754	0.99912

**Table 5:** GPC parameters.

Sample ID	M_p_ (g/mol)	M_n_ (g/mol)	M_w_ (g/mol)	PDI (-)
**Pen60/70**	917 ± 9	682 ± 27	2371 ± 575	3.4619 ± 0.7065
**Evotherm-DAT**	882 ± 14	513 ± 16	900 ± 10	1.3722 ± 0.4696
**HBO**	145 ± 4	193 ± 18	252 ± 40	1.3052 ± 0.0859
**AR**	866 ± 4	593 ± 13	2864 ± 622	4.8357 ± 0.9078
**ER-A**	873 ± 5	585 ± 4	2425 ± 737	4.1527 ± 1.2857
**BR-A**	896 ± 5	658 ± 4	1992 ± 6	3.0289 ± 0.0066
**AR-B**	877 ± 5	586 ± 12	2059 ± 374	3.5231 ± 0.7112
**ARB**	836 ± 3	496 ± 9	2535 ± 213	5.053 ± 0.4030

The numbers after “±” are standard deviations.
